# Crown-of-thorns starfish have true image forming vision

**DOI:** 10.1186/s12983-016-0174-9

**Published:** 2016-09-06

**Authors:** Ronald Petie, Anders Garm, Michael R. Hall

**Affiliations:** 1 Department of Biology, Marine Biological Section, University of Copenhagen, Universitetsparken 4, 2100 Copenhagen Ø, Denmark; 2 Australian Institute of Marine Science, PMB 3, Townsville MC, Townsville, 4810 QLD Australia

**Keywords:** *Acanthaster planci*, Sensory biology, Eyes, Orientation

## Abstract

**Background:**

Photoreceptors have evolved numerous times giving organisms the ability to detect light and respond to specific visual stimuli. Studies into the visual abilities of the Asteroidea (Echinodermata) have recently shown that species within this class have a more developed visual sense than previously thought and it has been demonstrated that starfish use visual information for orientation within their habitat. Whereas image forming eyes have been suggested for starfish, direct experimental proof of true spatial vision has not yet been obtained.

**Results:**

The behavioural response of the coral reef inhabiting crown-of-thorns starfish (*Acanthaster planci*) was tested in controlled aquarium experiments using an array of stimuli to examine their visual performance. We presented starfish with various black-and-white shapes against a mid-intensity grey background, designed such that the animals would need to possess true spatial vision to detect these shapes. Starfish responded to black-and-white rectangles, but no directional response was found to black-and-white circles, despite equal areas of black and white. Additionally, we confirmed that starfish were attracted to black circles on a white background when the visual angle is larger than 14°. When changing the grey tone of the largest circle from black to white, we found responses to contrasts of 0.5 and up. The starfish were attracted to the dark area’s of the visual stimuli and were found to be both attracted and repelled by the visual targets.

**Conclusions:**

For crown-of-thorns starfish, visual cues are essential for close range orientation towards objects, such as coral boulders, in the wild. These visually guided behaviours can be replicated in aquarium conditions. Our observation that crown-of-thorns starfish respond to black-and-white shapes on a mid-intensity grey background is the first direct proof of true spatial vision in starfish and in the phylum Echinodermata.

**Electronic supplementary material:**

The online version of this article (doi:10.1186/s12983-016-0174-9) contains supplementary material, which is available to authorized users.

## Background

Light sensitivity can be found in echinoderms like sea urchins (Echinoidea), sea cucumbers (Holothuroidea), starfish (Asteroidea) and brittle stars (Ophiuroidea) [1]. Sea urchins respond to shadows with movements of their spines [2, 3]. In addition, some sea urchins will cover themselves with objects in response to light [4], or display negative phototaxis. Even though sea urchins do not have eyes, species such as *Echinometra lucunter* L., *Echinometra viridis* and *Strongylocentrotus purpuratus* nevertheless orient towards visual targets and have been suggested to have a limited form of spatial vision, possibly by means of combining a dermal light sensitivity with shading by the spines [5, 6]. However, these sea urchin studies were examining orientational capabilities towards black circles on a light background; a stimulus that can be detected without using spatial resolution vision by following the gradient in light intensity. Other authors found that only certain regions of the sea urchin dermis were responsive to visual stimulation [2, 7] which could be explained by the relatively high opsin and pax 6 concentrations found in the tube feet of sea urchins [8, 9]. In addition, depressions in the skeleton of the sea urchin could provide the shading needed for directional sensitivity [10], providing an alternative hypotheses to the shading by the spines presented above.

Similarly, brittle stars have been found to change colour [11] in response to changes in illumination and display phototaxis [11, 12]. Morphological and optical investigations suggest that calcite structures in the epidermis of brittle stars [13] can be used to focus light onto putative light sensitive neurons. However, physiological and behavioural data proving light reception in these structures are still lacking [14].

Eyes have even been found on a sea cucumber, *Opheodesoma spectabilis*, and are associated with negative phototaxis [15]. The eyes are simple ocelli [16] and are thought to provide information about the intensity and direction of sunlight.

The starfish eye represents the most advanced light receptive structure in the echinoderm phylum and was first described more than 200 years ago by Vahl in 1780, cited by Smith [17]. The starfish eye has been described as the optic cushion, or terminal eye spot and arises from the first developing, primary podium [18, 19]. This results in one eye at the base of the terminal tube foot, at the tip of each and every arm. In starfish, tube feet have a diversity of functions and are responsible for adhesion [20], locomotion [21], respiration and secretion [22] and they are prominent sense organs that contain many sensory cells [23]. Starfish have been found to respond to mechanical [24] and olfactory stimulation [25, 26], both of which are senses that can augment vision during orientation tasks.

Some authors argue that calcite structures in the epidermis could provide starfish with a second eye-based visual system, similar to the one found in brittle stars. Present day starfish [27], as well as fossilised starfish [28], were described to have putative calcite lenses. However, in contrast to brittle stars, no neurons have been described to be associated with these putative lenses which making it problematic to assign function.

Starfish have also been reported to have extra-ocular light sensitivity using a dermal light sense. The starfish *Asterias amurensis* [29, 30] and *Asterias forbesi* [31] have been shown to exhibit phototactic movements in response to visual stimulation in both intact and blinded animals, demonstrating that eyes are not a requirement for photaxis and extra-ocular photoreception suffices. Dermal light sensitivity in starfish is less sensitive than vision using the eyes [30], which would make it ineffective at visual tasks requiring spatial resolution [32] and is therefore only likely to be involved in simple visual tasks like phototaxis.

Compound eyes have been found in many of the examined starfish species, however only recently the function of the compound eyes of starfish was revealed in the blue Star, *Linckia laevigata*, which was shown to orient towards coral reefs using their compound eyes [33]. Blinded starfish, with their extra-ocular photoreception and olfaction intact, were unable to navigate towards the reefs. Similar results have been obtained in the crown-of-thorns starfish, *Acanthaster planci* [34, 35]. With these findings in mind, it is clear that the system supporting more advanced visually guided behaviours in starfish is the compound eye.

The corallivorous crown-of-thorns starfish, is probably best known for exhibiting large population fluctuations. The abundance of this starfish can increase by six orders of magnitude within 1 to 2 years [36] and these outbreaks have been reported to be a major cause of coral mortality throughout the Indo-Pacific with flow-on ecosystem consequences [37–39]. Although much is known of their ecology, much less is known of their sensory biology and how this relates to their interaction with their environment. As has been reported in other starfish species [30, 33, 40] crown-of-thorns starfish have eyes and respond to visual stimulation [34, 35]. Each compound eye has on average 250 eye cups (ommatidia) for animals with a diameter of about 35 cm [34]. Each ommatidium contains two cell types: unpigmented photoreceptor cells and pigmented supportive cells that make up the pigment screen surrounding each ommatidium [33, 40]. The eye of the crown-of-thorns starfish is similar to the eye of *L. laevigata* [33], with the exception of the visual field which is flattened horizontally and measures approximately 100° wide and 30° high [34]. In addition, the spatial resolution of *A. planci* is better than the 16° found for *L. laevigata* and measures approximately 8°. The eye of the crown-of-thorns starfish is situated on a movable knob [41] which, compared to *L. laevigata*, increases the degree of control over the eye. *L. laevigata* lives on the same coral reefs as the crown-of-thorns starfish and has 5 arm, whereas the crown-of-thorns starfish has between 7 to 23 arms [42], which combined with the visual fields implies that both species have surround vision.

In this paper we set out to investigate which visual cues are used by the crown-of-thorns starfish for visual orientation. We present behavioural data from aquarium experiments, where the visual scene was controlled in detail. We tested whether the starfish use simple phototaxis or rely on true spatial vision for visual orientation tasks.

## Results and discussion

### Spatial vision

Orientation towards gradients in light intensity by means of phototaxis is the simplest form of directional photoreception [32, 43]. Phototaxis controls the simplest visually guided behaviours, requires the simplest systems for directional photoreception [32] and is, for instance, found in cnidarian larvae [44] and nematodes [45]. Two basic mechanisms can enable an organism to use light intensity distributions as orientation cues. Animals can use a sequence of samples from the environment (klinotaxis), or alternatively acquire information from receptor arrays, where each element in the array samples a different area in space (tropotaxis) [43] and information about the distribution of light is acquired instantaneously. In the latter case true spatial vision and image formation is implemented and this is what was tested for in the following experiments.

**Fig. 1:**
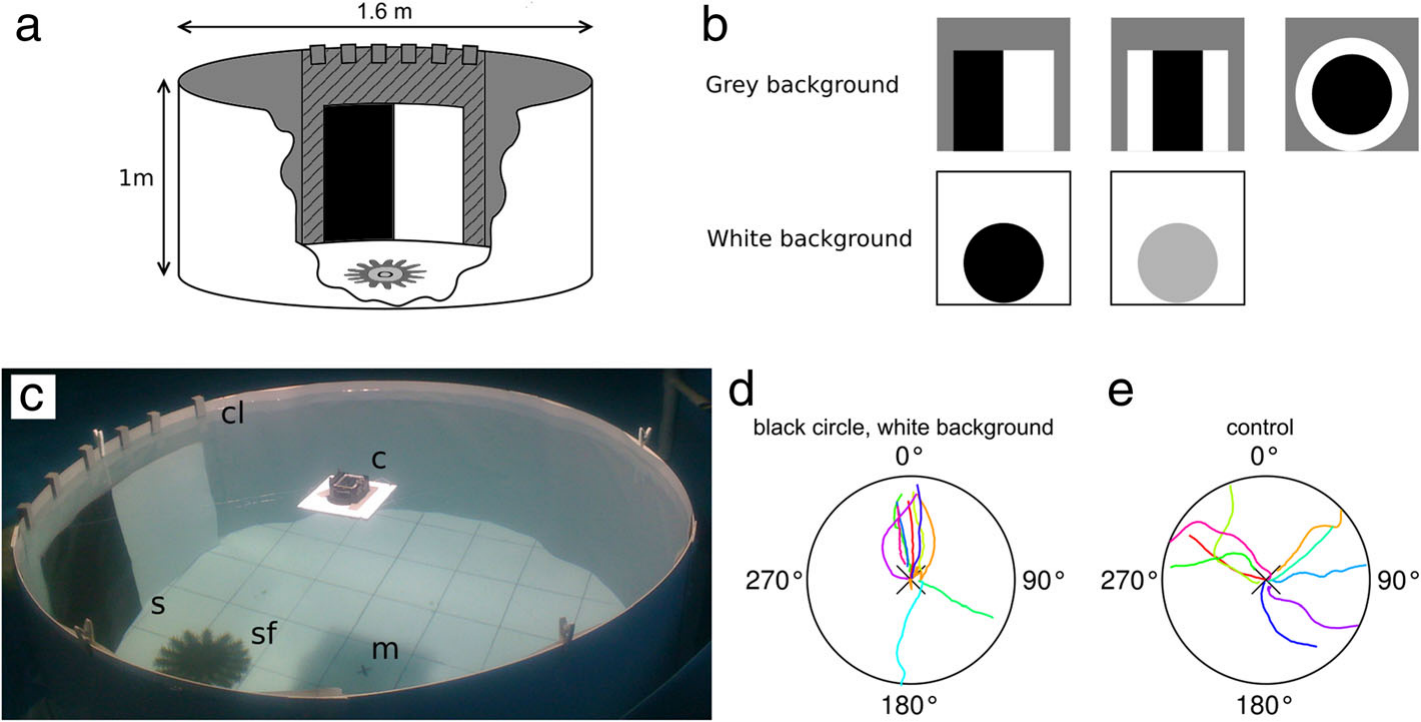
Behavioural arena. **a** Schematic representation of the behavioural arena. Visual stimuli were attached to a Plexiglas sheet (indicated by hatching) using Velcro, with the bottom of the stimulus on the floor of the arena. The sheet was lowered into the arena and fastened by clamps. The wall of the arena was white. For the experiments with black-and-white patterns, a mid-intensity grey cloth was attached to the inside wall of the arena. **b** Five different stimuli were presented to the animals: three black-and-white stimuli, a black circular stimulus and a grey circular stimulus. For each black-and-white stimulus the area of black was equal to the area of white. For all similar sized circular, or rectangular, stimuli the area of black was the equal. **c** The arena during an experiment. Recordings were made with a camera floating on the surface of the water. Abbreviations: c, camera; cl, clamps for attaching the Plexiglas sheet; m, middle of the arena; s, stimulus; sf, starfish. Example tracks for: **d** black circles (angular height 37°) on a white background and **e** the control experiment with only the Plexiglas sheet on a white background. The stimulus is located at 0°

**Fig. 2:**
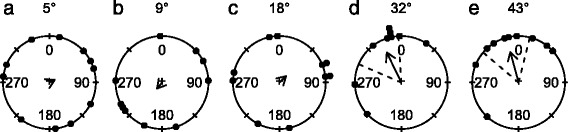
Behavioural results: paired black and white rectangles presented on the mid-intensity grey background. **a-e** Animals were attracted to stimulus when the angular height of the stimuli was 32° or larger. The stimuli were always presented with the black rectangle to the left. The mean vector of the significant responses is pointing left of 0°, indicating that on average the animals were headed to the black half of the stimulus. In the circular plots, the small, black, filled circles represent the final angular positions of the animals. The direction of the arrow indicates the mean direction and the length of the arrow represents the length of the mean vector (rho). The radius of the circle represents a vector length of 1. The 95 % confidence intervals for the response are indicated by dashed lines, only when the p-value for the Rayleigh test is smaller than 0.05. A summary of the circular statistics is given in Table 1

Detailed examinations of the visual capabilities of the crown-of-thorns starfish were conducted under controlled conditions in a circular behavioural arena (Fig. 1) which itself was situated in a large aquarium tank. The visual environment in the arena was designed such that the stimuli were detectable only by means of true spatial vision. We found that starfish were attracted to paired black and white rectangles on a grey background with 32° and 43° initial angular heights (Fig. 2, Table 1), while they did not respond to smaller stimuli. Note that in all circular diagrams only statistically significant mean headings are accompanied by the 95 % confidence intervals. Further details can be found in Table 1. The black rectangle was positioned left of the white one (See Fig. 1b) and animals were found to orient towards the black rectangle, shown by the mean vectors which were directed left of centre (Fig. 2d,e). This is the first direct proof that starfish use true spatial vision for the detection of visual targets, since the average intensity of light reflected off the stimuli was the same as the grey background. Additionally, it shows that crown-of-thorns starfish prefer to orient towards dark objects. This is in line with the observations on *A. planci* and *L. laevigata* that are attracted to coral reefs, which appear dark when filtered through the animals’ spectral sensitivity and spatial resolution [33, 34]. Starfish did not respond to the control stimulus, a transparent Plexiglas sheet, whether presented on a grey background (Fig. 4f) or a white background (Fig. 6f).

**Table 1:** Circular statistics summary. Rho denotes the relative length of the mean vector. Given p-values are for the Rayleigh test. For more information see text

Experiment	Background	Test type	Stimulus	N	Mean heading (°)	Rho	*p*-value
Paired rectangles	grey	angular	5°	11	63	0.13	0.839
	grey	angular	9°	10	220	0.17	0.763
	grey	angular	18°	10	46	0.20	0.682
	grey	angular	32°	9	334	0.72	<= 0.01
	grey	angular	43°	10	345	0.68	<= 0.01
Centred black rectangle	grey	axial	5°	10	314	0.67	<= 0.01
	grey	axial	9°	10	346	0.21	0.658
	grey	axial	18°	10	357	0.25	0.547
	grey	axial	32°	10	352	0.57	<= 0.05
	grey	axial	43°	10	5	0.80	<= 0.01
Black and white circle	grey	angular	4°/5°	9	193	0.32	0.402
	grey	angular	7°/10°	9	49	0.51	0.096
	grey	angular	14°/20°	9	28	0.36	0.331
	grey	angular	27°/35°	10	47	0.37	0.264
	grey	angular	37°/47°	9	60	0.41	0.23
	grey	angular	control	10	3	0.16	0.788
Black circle	white	angular	control	9	21	0.25	0.579
	white	angular	4°	8	48	0.15	0.837
	white	angular	7°	9	263	0.15	0.821
	white	angular	14°	9	334	0.65	<= 0.05
	white	angular	27°	9	348	0.79	<= 0.01
	white	angular	37°	10	5	0.66	<= 0.01
Grey circle	white	angular	0.1	10	286	0.02	0.997
	white	angular	0.3	10	22	0.42	0.168
	white	axial	0.5	10	6	0.63	<= 0.05
	white	angular	0.7	9	13	0.57	<= 0.05
	white	angular	0.9	10	5	0.94	<0.001

**Fig. 3:**
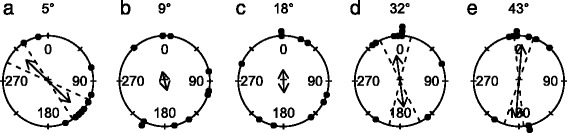
Behavioural results: black rectangle centred on a white square, presented on the mid-intensity grey background. **a-e** Starfish were either attracted to this stimulus or repelled, as seen by the axial nature of the directional responses. Animals responded to rectangles with an angular height of 5°, 32° and 43°. For more details on the circular plots see caption of Fig. 2. A summary of the circular statistics is given in Table 1

The response to black rectangles centred in a white square, presented on a grey background (Fig. 3, Table 1), resembled the response to the previous experiment, although, a high proportion of the animals moved away from the stimulus, resulting in an axially directed response. Animals responded to the smallest stimulus of 5° and the largest two of 32° and 43°. Axial responses occur more frequently in echinoderms and have also been observed in sea urchins [6], brittle stars [12] and other starfish [29]. The response to the 5° target cannot be readily explained. If the minimal object size that evokes a response really is 5°, it is to be expected that there would have been responses to all larger stimuli in the same experiment and other similar sized stimuli, but this was not observed. Regardless of whether the animals are repelled or attracted, the stimulus has to be visually detected, and hence this experiment also confirms that the crown-of-thorns starfish uses true spatial vision.

The axial nature of the responses observed could indicate a dual nature in response behaviours. We hypothesise that crown-of-thorns starfish are sometimes attracted to dark shapes as this is how their shelter and food source, the coral reef, would appear. However, dark shapes, especially moving ones, could also represent potential predators, which would need evasive or defensive action. Know predators of juvenile and adult crown-of-thorns starfish are: the triton snail, *Charonia tritonis* [46], the Maori wrasse, *Cheilinus undulates* [47, 48], damselfishes [49] and the vagabond butterfly fish, *Chaetodon vagabondus* (personal observations). The size of the starfish could be an important factor determining their behavioural response pattern, as small starfish are known to remain well hidden. Larger starfish appear to be less prone to predatory attack due to their array of sharp spines and appear more often fully exposed [50]. Small starfish could therefore be more attracted to dark hideouts than larger ones. However, we did not find any difference in response heading of all combined experiments when grouping the animals into progressively increasing 10 cm size bins (circular ANOVA, F_4,300_ = 0.87, *p* = 0.48), at least under aquarium conditions. It is possible that the animals’ previous experience in combination with its behavioural preference could influence the “motivational state” of the animal and therefore the response to the stimulus. A similar ambiguous behaviour can be found in small predators that need to decide whether an object is to be attacked or avoided [51, 52]. Making decisions to avoid visible objects can be mediated by the olfactory sense, as observed in sea urchins that are capable of distinguishing between a nearby active and inactive predator by using their sense of smell [53].

**Fig. 4:**
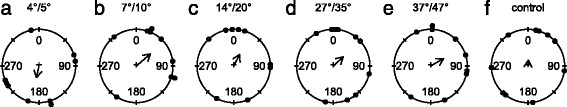
Behavioural results: black-and-white circles on a mid-intensity grey background. **a-f** Animals did not show directional responses to the circles or the control experiment, where the Plexiglas sheet was presented against the grey background. The angular height of the black centre of the stimulus is given first, followed by the angular height of the entire stimulus. For more details on the circular plots see caption of Fig. 2. A summary of the circular statistics is given in Table 1

**Fig. 5:**
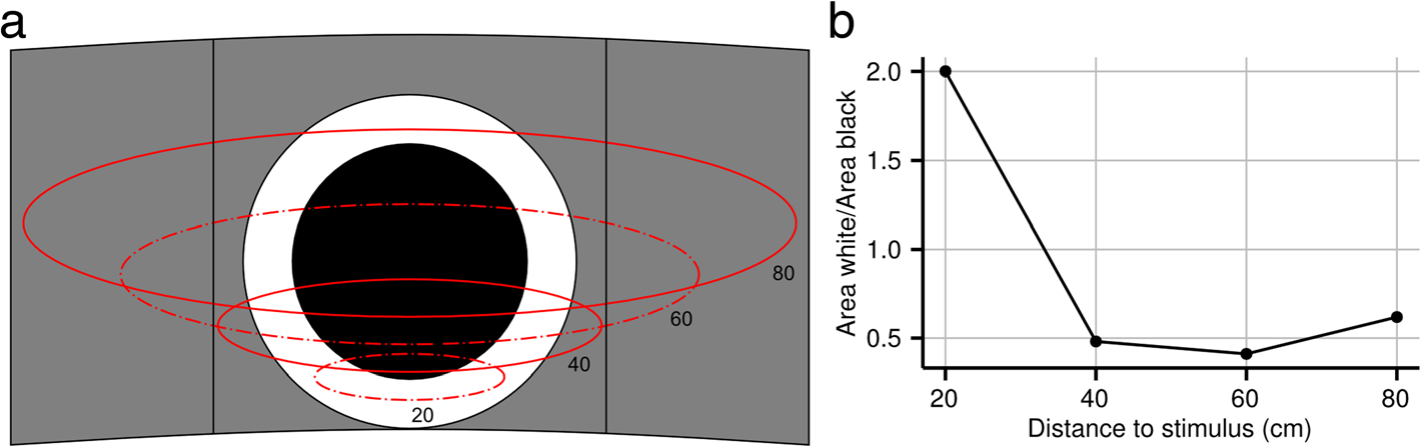
Crown-of-thorns starfish perception of the black-and-white circles at varying distances. **a** Visual representation of the stimulus and the field of view of a single eye at varying distance between the starfish and the stimulus. The numbers indicate the distance of the eye to the stimulus in centimetres and the red lines mark the outline of the visual field. The stimulus circle shown here has an angular height of 37° seen from the centre of the arena. **b** Relation between the proportion of white in the field of view and the distance to the stimulus. Note that at close distance to the stimulus, the white area sampled by the eye was twice the size of the sampled black area

Starfish were also presented with black-and-white circles against a grey background. Under these conditions the starfish did not show any directional response to the stimuli (Fig. 4), even though the area of black and white was equal to the previous two experiments. A possible explanation for the lack of response to the black-and-white circles on a grey background could be found in the white rim of the stimulus. The crown-of-thorns starfish has a narrow visual streak directed approximately 30° above the horizon [41]. As the animal moves from the centre of the arena, at a distance of 80 cm from the wall, to a position 20 cm in front of the stimulus, the relative area of white in the field of view of the eye directed towards the circle would increase in size four times (Fig. 5) compared to initial condition. Combining this with our observation that crown-of-thorns starfish prefers black over white it could imply that our circular stimulus gets increasingly unattractive as the animal moves closer.

### Response to black circles

**Fig. 6:**
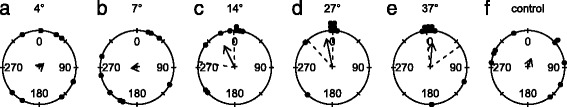
Behavioural results: black circle on a white background. **a-e** Starfish were attracted to circles with initial angular sizes of 14° or larger. All stimuli were attached to a Plexiglas sheet using Velcro, and the entire sheet was placed in the behavioural arena. **f** Starfish where not attracted to the control stimulus, the Plexiglas sheet alone, without stimulus patterns. For more details on the circular plots see caption of Fig. 2. A summary of the circular statistics is given in Table 1

Starfish were presented with stimuli with the highest possible contrast: black circles on a white background. Starfish were attracted to black circles when the angular size of the stimulus was 14° and larger (Fig. 6, Table 1). This indicates that the behavioural threshold for orientation towards these high contrast stimuli lies between an angular size of 7° and 14°. No directional response was observed for the control experiment (See Fig. 6f and Table 1). The behaviour is similar to that found in the sea urchins *E. lucunter* and *E. viridis* [5], which were attracted to circles with a minimal angular height of 33°. Interestingly, the sea urchin *S. purpuratus* showed an axial response to a smaller target than the crown-of-thorns starfish, a 10° black circle [6]. Testing the animals with black circles on a white background provided a means of comparing the behaviour of crown-of-thorns starfish with that observed previously in sea urchins, it does not, however, provide a measure for the lower behavioural threshold for spatial resolution in starfish. It is important to note that dark stimuli that are smaller than the resolution of the eye would still cause a decrease in light intensity in at least one ommatidium, which could be detected by the animal. The mean walking speed for crown-of-thorns starfish when presented with the 37° target was 22 cm/min (SD = 7.0, *N* = 10), which did not differ significantly from the 20 cm/min (SD = 6.2, *N* = 9) observed for the control experiment (*t*-test, *t* = 0.91, *p* = 0.38).

### Contrast sensitivity

**Fig. 7:**
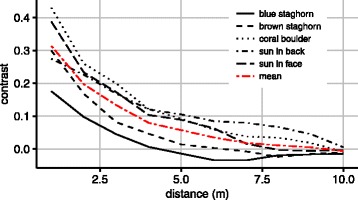
Contrasts between corals and the surrounding water within the coral reef environment. Contrasts were measured on images taken through a blue filter with a transmittance curve similar to the spectral sensitivity curve of the starfish eye. The contrast at 1 m measured 0.43 for the coral boulder and 0.18 for the blue staghorn coral. Two measurements were done on the same small reef, the first with the sun in the back and the second facing the sun

Contrasts were examined *in situ* within the coral reef environment on four different reefs and tested at different distances (Fig. 7). In general, contrast under water quickly diminishes with increasing distance [54]. The best contrast found was at 1 m distance and determined as 0.43 for coral boulders. The blue stag horn coral (*Acropora* sp.) had the lowest visual contrast at 1 m of only 0.18. It had a very similar colour [55] to the blue background and the branching growth pattern left a significant amount of background exposed which also decreases the contrast. Brown staghorn coral, which absorbs more blue light [55], had a slightly higher contrast. At 1 m distance the average contrast for all reefs was 0.31 (SD = 0.1, *N* = 5) which declined to 0.06 (SD = 0.05, *N* = 5) at 5 m. At 10 m the contrast was below detection levels of the camera.

**Fig. 8:**
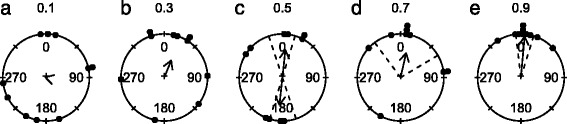
Behavioural results: grey circle on a white background. **a-e** Animals responded to circles with an initial angular size of 37° when the contrast was 0.5 or higher. For more details on the circular plots see caption of Fig. 2. A summary of the circular statistics is given in Table 1

The contrast sensitivity of starfish was assessed in aquarium experiments by presenting the animals with full sized circular stimuli (angular diameter: 37°) with decreasing contrast against a white background (Fig. 8, Table 1). We found that animals responded to stimuli with contrasts between 0.9 and 0.5, and were attracted to contrasts of 0.9 and 0.7, while they showed an axial response to stimuli with a contrast of 0.5. This implies that the behavioural threshold for contrast sensitivity lies somewhere between 0.3 and 0.5. It is unclear if the deviating response to contrasts of 0.5 has a functional relevance. As a comparison, cuttlefish are able to respond to contrast differences of only 0.15 under laboratory conditions [56] whereas humans have been found to detect contrast differences of just 0.005 [57]. It needs to be noted that in case of behavioural experiments with animals there can be a gap between the detection threshold and the behavioural threshold, whereas this is usually not the case in human experiments.

Crown-of-thorns starfish have been found to readily visually detect a coral reef when placed one meter in front of it [34]. At this distance the highest measured contrast of 0.43 is in the range of the contrast sensitivity threshold of between 0.3–0.5 observed in the behavioural arena. This indicates that the behaviours observed in the arena are similar to what is observed in the wild. The angular height of the grey stimulus circle is comparable to the 45° which a coral reef of 1 m would measure from a distance of 1 m. However, from a low benthic perspective a coral reef would usually provide a wider visual stimulus horizontally. A wider stimulus would likely be attractive at a lower contrast, since it would be visible to more of the eyes. If visual information is integrated in a manner similar to the mechanism proposed for olfaction [26], stimulating more eyes would result in more accurate orientation towards the stimulus. It could also explain why crown-of-thorns starfish so readily orient towards reefs at even lower contrasts. Future investigations on this aspect should focus on testing a greater range of stimuli widths and contrasts.

### Sensory biology

To date, the visual ecology of starfish has been primarily studied in the blue star [33] (*L. laevigata*) and the crown-of-thorns starfish [34, 35]. Both species inhabit a similar habitat and have comparable eyes. As smaller crown-of-thorns starfish (<30 cm) are reported to be cryptic during the day and more active during the night [50], the question arises whether this starfish can use visual cues at night. The blue star was reported to be unable to use vision on a starry, but moonless, night [33], which makes it plausible that the crown-of-thorns starfish is also unable to use vision at similar intensities. It would, however, be interesting to test the visual navigational capabilities of both species at slightly higher light intensities, such as light intensities up to full moon intensities. It is clear that both species can use visual orientation cues during the day, possibly to find their way back to the reef in case they have strayed off it during the night. Or, in case of the crown-of-thorns starfish, an individual could relocate the reef after it has been chased off by animals defending corals, such as guard crabs [58].

## Conclusions

Olfaction has been considered to be the singular dominant sensory modality in starfish [24, 59, 60], while it was assumed that any light guided behaviour would be restricted to simple phototaxis [29–31]. Our experiments provide proof for the use of true spatial vision for orientation, and show differences in response depending on the spatial pattern of the stimulus. Vision, however, is only going to be effective in close range detection of objects since visual contrast rapidly degrades over distance under water. Vision and olfaction likely complement each other, where olfaction would be much more effective over longer distances. As the starfish approaches a physical structure, which may have attracted it due to olfactory stimulation, vision would become the dominant cue since olfaction is less effective in the turbulent flow patterns that can occur around large objects at close range [43, 61].

## Methods

### Contrast measurements in the natural habitat

The contrast measurements were conducted on Lizard Island Research Station, Australia, on 10 and 11 August 2015. The contrast measurements were conducted around Horseshoe Reef (-14.687109 S, 145.444069 E) and in the lagoon directly south of Lizard Island (-14.694265, 145.454788 E). In order to measure the contrast between coral boulders and the surrounding water as perceived by the starfish, we placed a blue filter in front of the lens of a *GoPro Hero 4 SILVER* camera (San Mateo, California, USA). The blue filter was a *172 Lagoon Blue filter* (Lee filters, Hampshire, UK) with a transmittance curve closely matching the spectral sensitivity of the eye of the animal (Additional file 1: Figure S1). From 1 to 10 m distance, pictures of the reef were taken under water with 1 m intervals. This was done twice for an isolated small reef, once with the sun in the back and once with the sun in the face, for a large boulder coral and separately for blue and brown staghorn coral. The contrasts were calculated from the digital images by converting the colour images into 8-bit grey scale images in *ImageJ*. The contrast was calculated as Weber contrasts since this method is best suited for calculating contrasts of objects against a uniform background:

$$ Contrast=\frac{I_{background}-{I}_{stimulus}}{I_{background}} $$

where *I* is the measured light intensity. The average pixel value of the sea in the background was used as *I*_*background*_ and the average pixel value of the reef of interest as *I*_*stimulus*_. Each location was measured three times and contrast values were averaged.

### Behavioural experiments

#### Animals

Animals were collected from the Great Barrier Reef off the coast of Cairns, Australia, by the Australian Marine Park Tourist Operators (AMPTO) crown-of-thorns starfish control program, and transported to the Australian Institute of Marine science (AIMS) in Townsville, Australia. The average water temperatures at the collection sites ranged from an average of 27 °C in May to 23 °C in July. In the aquaria, the starfish were maintained in holding tanks with running, filtered seawater with a temperature of 24 °C and a salinity of 35‰. In total 72 starfish were used and some animals were used in two experiments (See Additional file 2: Table S1). The starfish had a mean diameter of 23 cm (min = 8, max = 43). The animals where not fed whilst held in the aquaria, but were used within on average 11 days of arriving at AIMS (min = 4, median = 6, max = 49). The starfish had between 12 and 20 arms (median = 16) and there was no difference in the number of arms, and thus the number of eyes per animal, between experiments (one way ANOVA, F_4,87_ = 0.56, *p* = 0.69).

#### Arena

The behavioural arena consisted of five white 1x1 m PVC sheets connected together to form a ring which had a circumference of 5 m, a diameter of 160 cm and a height of 1 m (Fig. 1a, c). The sheets were 3 mm thick and the water depth was 1 m. The arena was situated indoors in a 4 m diameter tank. The arena was lit from above with a full spectrum light emitting plasma (LEP) lamp (Model: GRE412R1C1WHC1101, Luxim, Sunnyvale, CA, USA). The light intensity in the arena centre, at the bottom, measured 2700 lux while it measured 2370 lux (SD = 105, *N* = 5) at the perimeter. Light intensities were measured using the luxmeter *amprobe lm-120* (Amprobe test tools Europe, Glottertal, Germany). The bottom of the arena consisted of a PVC plate with a 20 cm grid drawn onto it. The visual stimuli were attached to a see-through Plexiglas sheet using white Velcro. The stimuli were presented to the animals by securing the Plexiglas sheet to the arena wall with custom-made clamps, matching the background colour of the arena. Stimuli were presented in semi-random order and were positioned in the middle of one of the five PVC sheets of the arena, making sure that each stimulus was presented at each location for the same number of times.

#### Stimuli

Starfish were tested against a total five sets of stimuli (Fig. 1b) all of which were attached to a transparent Plexiglas sheet and placed with the lower edge on the arena floor. All stimuli and the mid-intensity grey background (discussed below), were printed at *Lotsa - Print & Signage* (Townsville, Australia) on a vinyl, water proof banner. The simplest stimuli used were black circles on a white background. The circles had angular heights of 4°, 7°, 14°, 27° and 37°, seen from the middle of the arena. In addition we presented a control stimulus consisting only of the Plexiglas sheet without a stimulus pattern. For the contrast sensitivity experiment five 37° high circles with different grey tones were presented against a white background. The contrasts of the circles were calculated as described above and were: 0.1, 0.3, 0.5, 0.7 and 0.9.

Additionally, three different black-and-white stimuli were presented against a mid-intensity grey background which had a reflected light intensity exactly between those found for black and white. Viewed under the light source used in the experiments, the reflected light intensity measured 105 % (SD = 1.04, *N* = 5) of the real mid-intensity grey value. The three stimuli were: a black rectangle next to a white rectangle, a black rectangle centred inside a white square, and a black circle inside a white circle (Fig. 1b). In all of the black-and-white patterns the area of white was equal to the area of black, which made the intensity of light reflected off the entire stimulus equal to the mid-intensity grey background. The size of the black-and-white stimuli was chosen such that the black part had the same area as the purely black circles. For the paired black and white rectangles, the black rectangle was always presented left of the white.

#### Protocol

Aquaria experiments were conducted between May and August 2015. After the stimulus was placed on one of five evenly spaced stimulus locations on the arena wall, a starfish was collected from the holding tanks and placed in the middle of the arena (Fig. 1). It was positioned with the oral side down and allowed to move freely. When the animal touched the arena wall the location was recorded. The angle between: (1) the stimulus, (2) the centre of the arena and (3) the animals’ final location was taken as the heading of the response. By measuring the angle in this way, the recorded response angle does not depend on the position where the stimulus was placed. Each animal was presented with a maximum of three stimuli, after which it wasn’t used for that experiment again. If animals were used again in another experiment they were allowed at least one day rest.

### Data recording and analysis

The response was recorded from 1 m height at the water surface using the *GoPro* camera inside a dive housing (Fig. 1c). An *Inon UFL-G140* fish eye lens (Kamakura, Japan) was used to enable us to capture the entire arena floor. The lens was mounted onto an *Inon SD Mount Cage* and the *GoPro* camera was placed inside this mount cage. The camera floated on the surface using a Styrofoam float, and was centred in the arena using transparent fishing line. Since the LEP lamp was also centred the float unavoidably casts a shadow on the bottom of the arena (See Fig. 1c). The control experiments (Figs. 1d, 4f and 6f), show that the animals do not use this shadow as an orientation cue.

The behaviour of the animals was recorded as a time-lapse series with a five second interval between the images. Image sequences were manually analysed using *ImageJ* (version: 1.47n) and the resulting data was analysed in *RStudio* (version: 0.98.1103) using custom written scripts for *R* (version 3.2.2) using the packages: *circular, dplyr, ggplot2, knitr* and *tidyr*.

### Statistical analysis

The directionality of the data was tested using the Rayleigh test from the R package *circular*. In the Rayleigh test, the null hypothesis tests for a random distribution of headings and the alternative hypothesis for a non-random distribution of the headings. Applying the Rayleigh test to the original headings tests for angular directionality in the data. By multiplying the original headings by two, followed by a Rayleigh test, axial directionality of the data was tested instead. All t-tests were confirmed by a Mann-Whitney test and all ANOVA’s by a Kruskall-Wallis test. The threshold for significance was set to 5 %.

## Additional file

Additional file 1: Figure S1. Spectral transmittance curve blue filter. The relative transmittance of the filter is drawn in blue and the spectral sensitivity of the crown-of-thorn starfish photoreceptors in black (taken from [34]). Grey shading indicating the standard deviation. (PDF 7 kb) Additional file 2: Table S1. Starfish usage data. (DOC 23 kb)
