# Supplementary figures and images for: Crown-of-thorns starfish have true image forming vision
Source: Front Zool. 2016 Sep 6;13(1):41. doi: 10.1186/s12983-016-0174-9 (PMC5013567; doi:10.1186/s12983-016-0174-9)

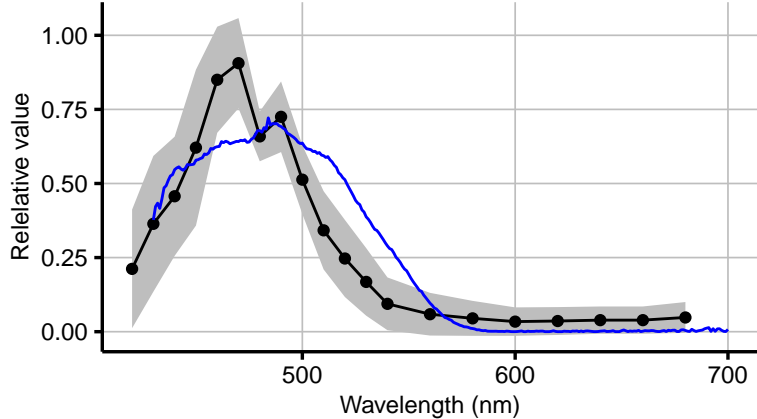

Supplement: Additional file 1: Figure S1. — Spectral transmittance curve blue filter. The relative transmittance of the filter is drawn in blue and the spectral sensitivity of the crown-of-thorn starfish photoreceptors in black (taken from [34]). Grey shading indicating the standard deviation. (PDF 7 kb) [file 12983_2016_174_MOESM1_ESM.pdf]
