# Supplementary material for: Crown-of-thorns starfish have true image forming vision
Source: Front Zool. 2016 Sep 6;13(1):41. doi: 10.1186/s12983-016-0174-9 (PMC5013567; doi:10.1186/s12983-016-0174-9)
Supplement: Additional file 2: Table S1. — Starfish usage data. (DOC 23 kb) [file 12983_2016_174_MOESM2_ESM.doc]

Supplementary tables

Ronald Petie^1^, Anders Garm^1^, Michael R. Hall^2^

Supplementary table 1. Starfish usage data

|  | days spent in captivity | | | | | |  | | animal usage | |
| --- | --- | --- | --- | --- | --- | --- | --- | --- | --- | --- |
|  | min | 1st quartile | median | 3rd quartile | max |  | | first use | | second use |
| Black circle on white | 4 | 5.2 | 6.5 | 16.8 | 21 |  | | 18 | | 0 |
| B&W circle on grey | 4 | 12.0 | 13.0 | 14.2 | 18 |  | | 11 | | 9 |
| Centred black rectangles | 4 | 4.8 | 5.0 | 6.0 | 6 |  | | 18 | | 2 |
| Grey circle on white | 4 | 5.0 | 5.0 | 6.0 | 8 |  | | 17 | | 0 |
| Paired rectangles | 6 | 11.0 | 21.0 | 26.0 | 49 |  | | 7 | | 9 |
